# Efficacy and safety of anti-PD-1 inhibitor versus anti-PD-L1 inhibitor in first-line treatment of extensive-stage small cell lung cancer: a multicenter retrospective study

**DOI:** 10.1186/s12885-024-11833-6

**Published:** 2024-01-17

**Authors:** Boyu Qin, Lingli Xin, Chen Liang, Lingling Li, Qi Song, Yaping Long, Xiaoling Zhang, Dan Wang, Weiwei Shi, Jing Zhang, Yi Hu, Bo Yang, Qi Xiong

**Affiliations:** 1 Senior Department of Oncology, The Fifth Medical Center of PLA General Hospital, 4th West Ring Road 100, Fengtai district, 100039 Beijing, China; 2 Department of Gynaecology and Obstetrics, PLA Rocket Force Characteristic Medical Center, Xinjiekou outer Street 16, Xicheng district, 100088 Beijing, China; 3 Department of Graduate Administration, PLA General Hospital, Fuxing Road 28, Haidian district, 100853 Beijing, China; 4 Medical Service Department, PLA General Hospital, Fuxing Road 28, Haidian district, 100853 Beijing, China; 5 Department of Oncology, The First Medical Center of PLA General Hospital, Fuxing Road 28, Haidian district, 100853 Beijing, China

**Keywords:** Small cell lung cancer, Anti-PD-1/PD-L1, Immunotherapy, Locoregional thoracic radiotherapy, Lactate dehydrogenase

## Abstract

**Background:**

Immunotherapy targeting PD-1/PD-L1 has revolutionized the treatment of extensive-stage small cell lung cancer (ES-SCLC). However, clinical trials suggest differential efficacy of anti-PD-1 agents and anti-PD-L1 agents in first-line treatment of ES-SCLC. This retrospective multicenter study aimed to compare the efficacy and safety of anti-PD-1 agents versus anti-PD-L1 agents in first-line treatment of ES-SCLC in real-world practice.

**Methods:**

Patients with pathologically or cytologically confirmed ES-SCLC treated with platinum plus etoposide combined with anti-PD-1 or PD-L1 agents as first-line treatment in different centers of PLA General Hospital between January 2017 and October 2021 were included for this study. Survival outcomes and safety were compared between patients receiving anti-PD-1 and PD-L1 agents.

**Results:**

Of the total 154 included patients, 68 received anti-PD-1 agents plus chemotherapy (PD-1 group), and 86 received anti-PD-L1 agents plus chemotherapy (PD-L1 group). Progression-free survival (PFS) and overall survival (OS) in the entire cohort were 7.6 months (95% confidence interval [CI]: 6.5–8.2 months) and 17.4 months (95% CI: 15.3–19.3 months), respectively. Median PFS and OS were comparable between the PD-1 group and PD-L1 group (PFS: 7.6 months vs. 8.3 months, HR = 1.13, 95% CI: 0.79–1.62, *p* = 0.415; OS: 26.9 months vs. 25.6 months, HR = 0.96, 95% CI: 0.63–1.47, *p* = 0.859. The objective response rate and disease control rate were comparable between the two groups: 79.4% vs. 79.1% and 92.6% vs. 94.2%, respectively. The 6-month, 12-month, and 18-month PFS and OS rates were slightly higher in the PD-L1 group than in the PD-1 group, while the 24-month PFS rate was slightly higher in the PD-1 group than in the PD-L1 group. Stratified analysis showed that locoregional thoracic radiotherapy and normal lactate dehydrogenase level were independent predictors of better OS in ES-SCLC patients treated with first-line chemotherapy plus ICI. Adverse events were not significantly different between the two groups.

**Conclusions:**

Anti-PD-1 agents and anti-PD-L1 agents combined with chemotherapy as first-line treatment for ES-SCLC are comparably effective and well tolerated.

**Supplementary Information:**

The online version contains supplementary material available at 10.1186/s12885-024-11833-6.

## Introduction

Small-cell lung cancer (SCLC) is a highly aggressive malignancy that accounts for about 15% of all lung cancers, the second most common malignancy worldwide and the commonest cause of cancer mortality in both sexes [1, 2]. About 80–85% of patients with SCLC have extensive-stage (ES) disease at diagnosis. For the last three decades, platinum-based doublet chemotherapy has been standard-of-care first-line treatment for ES-SCLC. Although the response rate to first-line chemotherapy is up to 80%, the prognosis of ES-SCLC is generally dismal, with median overall survival (OS) being less than 1 year and fewer than 5% of patients surviving beyond 2 years [2, 3]. Thus, novel treatments are urgently needed for ES-SCLC.

The introduction of immune checkpoint inhibitors (ICIs) targeting the programmed cell death protein-1 (PD-1) or programmed death protein ligand-1 (PD-L1) have greatly changed the treatments of non-small cell lung cancer (NSCLC), malignant melanoma, and renal and other cancers. ICIs might potentially be effective in SCLC, which are immunogenic with high likelihood of tobacco-induced mutations [4]. Several clinical trials have shown that anti-PD-1/PD-L1 agents have promising efficacy as later-line therapy for SCLC [5–7]. Moreover, the anti-tumor efficacy of immunotherapy appears to be potentiated by chemotherapy [8]. Therefore, it would be rational to combine anti-PD-1/PD-L1 agents with chemotherapy for first-line treatment of ES-SCLC. However, the results of previous studies were not consistent. In the IMpower133, CASPIAN, and CAPSTONE-1 studies, ES-SCLC patients receiving first-line treatment with anti-PD-L1 agents (atezolizumab/durvalumab/adebrelimab) plus standard chemotherapy followed by maintenance anti-PD-L1 agent had significantly better OS than patients receiving chemotherapy alone [9–12]. In contrast, in the CheckMate-451 study, first-line chemotherapy followed by maintenance with anti-PD-1 agent (nivolumab) failed to prolonged OS in ES-SCLC [13]. Furthermore, in the KEYNOTE-604 study, pembrolizumab, another anti-PD-1 agent, combined with chemotherapy as first-line treatment, failed to significantly prolong OS in ES-SCLC [14]. Meanwhile, the recent ASTRUM-005 study showed that first-line treatment with chemotherapy plus the anti-PD-1 agent serplulimab significantly extended OS and reduced death risk by 38% in ES-SCLC [15]. Although these findings suggest differential efficacy between anti-PD-1 agents and anti-PD-L1 agents in ES-SCLC, a meta-analysis found no difference in clinical efficacy between anti-PD-1 agents and anti-PD-L1 agents [16].

Due to the rigorous inclusion criteria of clinical trials, the characteristics of enrolled patients are very different from those encountered in real-world practice, leading to real-world evidence as mutually complementary for clinical trials [17]. To the best of our knowledge, there is no study based on real-world data that directly compares efficacy between anti-PD-1 agents and anti-PD-L1 agents in ES-SCLC. Therefore, in this retrospective study based on real-world data from four centers, we aimed to compare the efficacy and safety of anti-PD-1 agents versus anti-PD-L1 agents in first-line treatment of ES-SCLC.

## Methods

### Patients

The study population comprised patients with ES-SCLC per Veterans Administration Lung Study Group staging system who had received first-line treatment with the combination of platinum plus etoposide and anti-PD-1 or PD-L1 agents at different centers (the first, third, fourth, and fifth medical centers) of the PLA General Hospital between January 2017 and October 2021. The inclusion criteria were as follows: pathologically or cytologically confirmed ES-SCLC; treated with at least one cycle of anti-PD-1 or anti-PD-L1 agents plus chemotherapy; with at least one measurable lesion; with clear prognostic information. Exclusion criteria were as follows: limited stage-SCLC; failed to be followed up; incomplete medical data; receiving platinum-free chemotherapy; receiving chemotherapy or immunotherapy alone; no measurable lesion. The patients were divided into PD-1 group and PD-L1 group according to the ICI received. The demographic and medical data of patients (sex, age, smoking history, Eastern Cooperative Oncology Group performance status (ECOG-PS), treatment regimen, brain/liver/bone metastases, radiotherapy (determined by radiation therapist when chemotherapy finished), radiological and laboratory test results) were collected from inpatient electronic patient record system.

### Treatments

Etoposide was administered at 100 mg/m^2^ for 3 consecutive days every 21-day cycle. Choice of the platinum agent—cisplatin (70–75 mg/m^2^), carboplatin (area under the curve = 4–5), or lobaplatin (30 mg/m^2^)—was at discretion of the treating physician and was based on the National Comprehensive Cancer Network guidelines or the Chinese Society of Clinical Oncology guidelines and the patient’s performance status. The number of cycles (four or six) and dosage of chemotherapy were determined by the treating physician according to the treatment response and the patient’s tolerance, and performance status. The anti-PD-1 agents—pembrolizumab (200 mg), nivolumab (200 mg), camrelizumab (200 mg), sintilimab (200 mg), and toripalimab (240 mg)—and anti-PD-L1 agents—atezolizumab (1200 mg) and durvalumab (1500 mg)— were administered every 21-day cycle and continued for a total of 2 years unless disease progression or unacceptable toxicity developed or the patient died. The last follow-up date was 10th February, 2023.

### Outcomes and evaluation

Tumor evaluation was performed at baseline, every 6 weeks for the first 24 weeks, and every 12 weeks thereafter until disease progression or treatment discontinuation. Progression free survival (PFS) was defined as the time from anti-PD-1/PD-L1 agent initiation until disease progression or death due any cause. OS was defined as the time from the initiation of the anti-PD-1/PD-L1 agent to death. Survival outcomes were assessed every 3 months after treatment discontinuation through phone call or outpatient service.

Tumor response was evaluated per Response Evaluation Criteria in Solid Tumors (RESICT) version 1.1, using radiological data and was categorized as complete response (CR), partial response (PR), stable disease (SD), or progressive disease (PD). The objective response rate (ORR) was the total percentage of patients achieving CR and PR, and the disease control rate (DCR) was the total percentage achieving CR, PR, and SD. Treatment-related adverse events (TRAEs) were graded using Common Terminology Criteria for Adverse Events (version 5.0).

### Statistical analysis

Categorical variables were summarized as frequency and percentage, and continuous variables as median with 95% confident interval (CI). The Pearson chi-square test and Fisher exact test were used to compare differences between the two groups. ANOVA was performed to compare differences among three or more subgroups. PFS and OS were estimated by the Kaplan–Meier method. The log-rank test was used for univariate analysis between groups. Factors significantly associated (at *p* < 0.05) with survival outcomes in univariate analysis were imported into Cox regression analysis to identify the independent risk factors for poor survival. Stratified analysis was performed to compare efficacy of the two ICIs in different subgroups. Strata with fewer than 10 events were excluded from stratified analysis. Statistical analysis was conducted using PRISM 7.0 (GraphPad Software, La Jolla, CA, USA) and SPSS 26.0 (IBM Corp., Armonk, NY, USA). Statistical significance was defined as *p* < 0.05 (two-sided).

## Results

### Baseline characteristics of patients

**Fig. 1:**
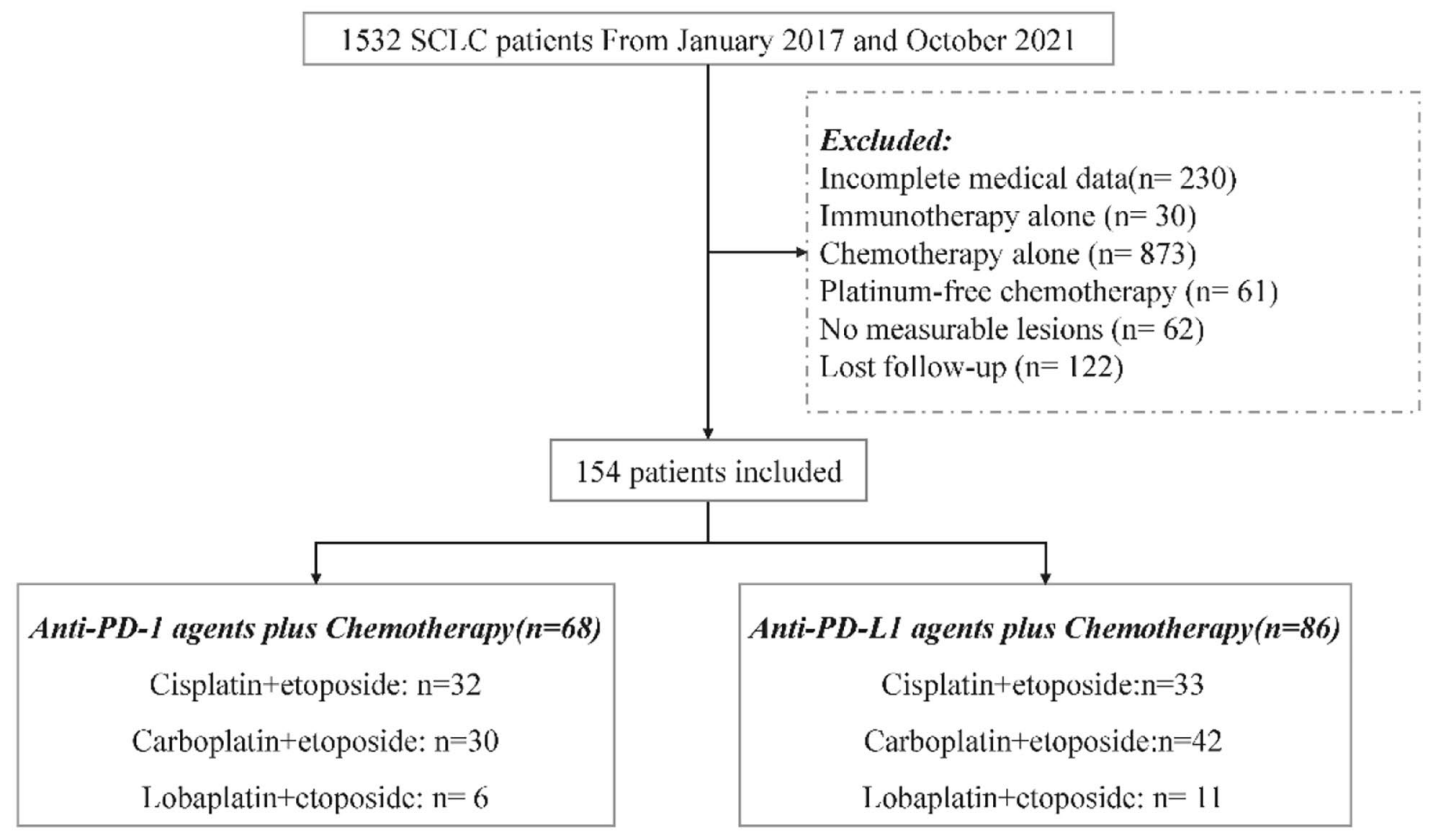
Workflow of this study

Out of 1532 patients with SCLC treated at four medical centers of PLA General Hospital between January 2017 and October 2021, 154 were eligible for our analysis (Fig. 1). Among these 154 patients, 87.7% were male, 66.2% were aged ≤ 65 years, 90.0% had ECOG-PS ≤ 1, 71.4% had history of smoking, 18.8% had liver metastases, and 16.2% had brain metastases. A total of 65, 72, and 17 patients, respectively, were treated with cisplatin, carboplatin, and lobaplatin. While, 68 patients received anti-PD-1 agents plus chemotherapy, 86 received anti-PD-L1 agents plus chemotherapy. Of the 86 patients in PD-L1 group, there were 27 patients receiving atezolizumab and 59 patients receiving durvalumab; of the 68 patients in the PD-1 group, 33 patients were treated with sintilimab, 14 with pembrolizumab, 12 with toripalimab, 6 with nivolumab, and 3 with camrelizumab. All patients received at least four cycles of chemotherapy combined with ICI. While 58.8% (40/68) patients received six cycles in the PD-1 group, 72.1% (62/86) received six cycles in the PD-L1 group. Two-year ICI treatment was completed by 7.4% (5/68) and 8.1% (7/86) patients in the PD-1 and PD-L1 groups, respectively. Locoregional thoracic radiotherapy was administered to 42.6% (29/68) and 46.5% (40/86) patients in the PD-1 group and PD-L1 group, respectively. Serum lactate dehydrogenase (LDH) level was < 250 U/L (reference value: 40–250 U/L) in 82/154 (53.2%) patients (39 in the PD-1 group and 43 in the PD-L1 group). Baseline clinical characteristics were not significantly different between both groups (Table 1).

**Table 1:** Demographics and baseline characteristics of patients included

	Total (*n* = 154)	PD-1 group (*n* = 68)	PD-L1 group (*n* = 86)	χ^2^	*p* value
Sex (%)				0.631	0.427
Male	135 (87.7)	58 (85.3)	77 (89.5)		
Female	19 (12.3)	10 (14.7)	9 (10.5)		
Age (%)				2.898	0.089
≤ 65	102 (66.2)	50 (73.5)	52 (60.5)		
> 65	52 (33.8)	18 (26.5)	34 (39.5)		
Liver metastases (%)				1.758	0.185
No	125 (81.2)	52 (76.5)	73 (84.9)		
Yes	29 (18.8)	16 (23.5)	13 (15.1)		
Brain metastases (%)				0.745	0.388
No	129 (83.8)	55 (80.9)	74 (86)		
Yes	25 (16.2)	13 (19.1)	12 (14)		
Bone metastases (%)				0.134	0.714
No	111 (72.1)	48 (70.6)	63 (73.3)		
Yes	43 (27.9)	20 (29.4)	23 (26.7)		
Smoking history (%)				1.646	0.200
No	44 (28.6)	23 (33.8)	21 (24.4)		
Yes	110 (71.4)	45 (66.2)	65 (75.6)		
ECOG PS (%)				0.011	0.918
≤ 1	140 (90.9)	62 (91.2)	78 (90.7)		
> 1	14 (9.1)	6 (8.8)	8 (9.3)		
Cycles of chemotherapy plus ICI					
4	52 (33.8)	28 (41.2)	24 (27.9)	2.426	0.119
6	102 (66.2)	40 (58.8)	62 (72.1)		
chemotherapy regimen (%)				1.401	0.496
EP	65 (42.2)	32 (47.1)	33 (38.4)		
EC	72 (46.8)	30 (44.1)	42 (48.8)		
EL	17 (11.0)	6 (8.8)	11 (12.8)		
immunotherapy regimen (%)					
pembrolizumab		14 (20.6)	-		
nivolumab		6 (8.8)	-		
sintilimab		33 (48.5)	-		
toripalimab		12 (17.6)	-		
camrelizumab		3 (4.5)	-		
atezolizumab		-	27 (31.4)		
durvalumab		-	59 (68.6)		
Locoregional thoracic radiotherapy(%)				0.229	0.632
No	85 (55.2)	39 (57.4)	46 (53.5)		
Yes	69 (44.8)	29 (42.6)	40 (46.5)		
LDH(%)				0.825	0.364
≤ 250	82 (53.2)	39 (57.4)	43 (50.0)		
>250	72 (46.8)	29 (42.6)	43 (50.0)		

EP: etoposide + cisplatin; EC: etoposide + carboplatin; EL: etoposide + lobaplatin. LDH: lactate dehydrogenase

### Efficacy and safety of ICI treatment in PD-1 group vs. PD-L1 group

**Fig. 2:**
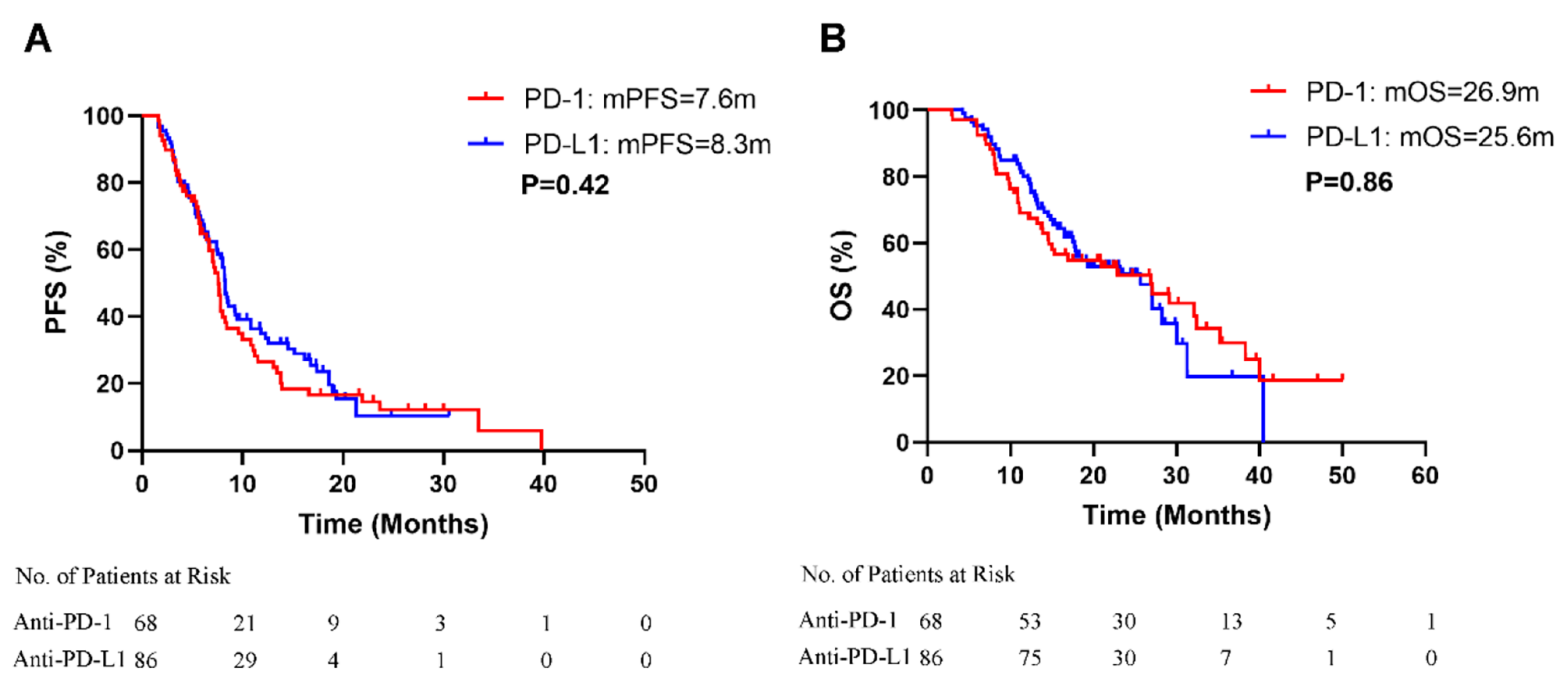
Kaplan-Meier curve of PFS and OS. **A**: PFS between PD-1 group and PD-L1 group; **B**: OS between PD-1 group and PD-L1 group

Median follow-up time was 24.7 months (range: 3.0-50.7 months). At the data collection cutoff date, 58.8% (40/68) patients had died in the PD-1 group and 53.5% (46/86) in the PD-L1 group. In the PD-1 group, 1 patient achieved CR and 53 achieved PR. In the PD-L1 group, no patient achieved CR and 68 patients achieved PR. The ORR (PD-1 group vs. PD-L1 group: 79.4% vs. 79.1%) and DCR (PD-1 group vs. PD-L1 group: 92.6% vs. 94.2%) were similar between the groups. The 6-month, 12-month, and 18-month PFS and OS rates were slightly higher in the PD-L1 group, while the 24-month PFS rate was marginally higher in the PD-1 group. However, there were no significant differences in median PFS (PD-1 group vs. PD-L1 group: 7.6 months vs. 8.3 months, HR = 1.13, 95% CI: 0.79–1.62, *p* = 0.415) and OS (PD-1 group vs. PD-L1 group: 26.9 months vs. 25.6 months, HR = 0.96, 95% CI: 0.63–1.47, *p* = 0.859) between the groups (Table 2; Fig. 2A-B).

**Table 2:** Comparison of efficacy between PD-1 group and PD-L1 group

	PD-1 group	PD-L1 group	*p* value
**CR**	1	0	
**PR**	53	68	
**SD**	9	13	
**PD**	5	5	
**ORR (%)(CR + PR)**	54 (79.4)	68 (79.1)	0.959
**DCR (%) (CR + PR + SD)**	63 (92.6)	81 (94.2)	0.956
**PFS**			
Events, n (%)	56 (82.4)	65 (75.6)	
Median, months (95%CI)	7.6 (6.936–8.264)	8.3 (7.717–8.883)	0.415
6-month rate (95%CI)	64.7% (53.1–76.3)	67.4% (57.4–77.4)	0.853
12-month rate (95%CI)	26.6% (15.4–37.8)	34.9% (24.3–45.5)	0.345
18-month rate (95%CI)	16.6% (7.2–26.0)	23.7% (13.7–33.7)	0.376
24-month rate (95%CI)	12.1% (3.5–20.7)	10.4% (0.0-20.8)	0.997
**OS**			
Events, n (%)	40 (58.8)	46 (53.5)	
Median, months (95%CI)	26.9 (13.805–39.995)	25.6 (16.214–34.986)	0.859
6-month rate (95%CI)	92.6% (86.3–98.9)	95.3% (90.8–99.8)	0.716
12-month rate (95%CI)	69.0% (58.0–80.0)	80.1% (71.7–88.5)	0.161
18-month rate (95%CI)	54.9% (42.9–66.9)	56.2% (45.4–67.0)	0.992
24-month rate (95%CI)	50.5% (38.0–63.0)	50.7% (39.1–62.3)	0.985

ORR: objective response rate, DCR: disease control rate, PFS: progression free survival, OS: overall survival

#### Stratified analysis

Stratified analysis was performed to identify patients most likely to benefit from anti-PD-1 or anti-PD-L1 agents. Among nonsmokers, PFS was significantly longer with PD-L1 treatment than with PD-1 treatment (16.2 months vs. 7.6 months, *p* = 0.047); however, OS was similar with both treatments. No statistically significant differences were found in PFS and OS between patients treated with PD-1 and PD-L1 in different subgroups of age, metastases site, chemotherapy agents, radiotherapy, and LDH levels (Supplemental Table 1).

#### Safety

One or more TRAEs occurred in 89.7% (61/68) patients in the PD-1 group vs. 94.2% (81/86) patients in the PD-L1 group; the difference was not statistically significant (Table 3). The most common TRAEs of any grade were myelosuppression, liver injury, hypopotassemia, and gastrointestinal reaction. The most common grade 3 or worse TRAEs in both groups were decreased white blood cell, anemia, and decreased platelet. Notably, 4.4% (3/68) patients in the PD-1 group and 8.1% (7/86) patients in the PD-L1 group experienced grade 3 pneumonia and needed discontinuation of ICIs. All received corticosteroid treatment, and the pneumonia resolved eventually. No treatment-related deaths occurred in either group.

**Table 3:** Safety Analysis between PD-1 group and PD-L1 group

Adverse event	All patients (*n* = 154)		PD-1 group (*n* = 68)		PD-L1 group (*n* = 86)		*p* value	
	Any grade (%)	≥ 3 grade (%)	Any grade (%)	≥ 3 grade (%)	Any grade (%)	≥ 3 grade (%)	Any grade (%)	≥ 3 grade (%)
White blood cell decreased	93 (60.4)	28 (18.2)	36 (52.9)	11 (16.2)	57 (66.3)	17 (19.8)	0.093	0.566
Anemia	96 (62.3)	13 (8.4)	41 (60.3)	8 (11.8)	55 (64)	5 (5.8)	0.642	0.187
Platelet count decreased	28 (18.2)	13 (8.4)	13 (19.1)	6 (8.8)	15 (17.4)	7 (8.1)	0.789	0.879
Liver damage	56 (36.4)	8 (5.2)	21 (30.9)	2 (2.9)	35 (40.7)	6 (7)	0.209	0.450
Total bilirubin increased	22 (14.3)	0 (0)	8 (11.8)	0 (0)	14 (16.3)	0 (0)	0.427	-
Direct bilirubin increased	16 (10.4)	2 (1.3)	6 (8.8)	0 (0)	10 (11.6)	2 (2.3)	0.571	0.504
Renal injury	15 (9.7)	1 (0.6)	6 (8.8)	1 (1.5)	9 (10.5)	0 (0)	0.733	0.442
Creatine kinase increased	18 (11.7)	2 (1.3)	5 (7.4)	1 (1.5)	13 (15.1)	1 (1.2)	0.136	1.000
Hypopotassemia	50 (32.5)	8 (5.2)	24 (35.3)	6 (8.8)	26 (30.2)	2 (2.3)	0.505	0.150
Amylase increased	6 (3.9)	0 (0)	1 (1.5)	0 (0)	5 (5.8)	0 (0)	0.167	-
Lipase increased	11 (7.1)	4 (2.6)	3 (4.4)	1 (1.5)	8 (9.3)	3 (3.5)	0.392	0.786
Pneumonia	15 (9.7)	10 (6.5)	7 (10.3)	3 (4.4)	8 (9.3)	7 (8.1)	0.837	0.547
Peripheral neuritis	2 (1.3)	1 (0.6)	2 (2.9)	1 (1.5)	0 (0)	0 (0)	0.193	0.442
Rash	8 (5.2)	0 (0)	5 (7.4)	0 (0)	3 (3.5)	0 (0)	0.479	-
Gastrointestinal reaction	50 (32.5)	1 (0.6)	21 (30.9)	0 (0)	29 (33.7)	1 (1.2)	0.709	1.000
Fatigue	11 (7.1)	1 (0.6)	2 (2.9)	0 (0)	9 (10.5)	1 (1.2)	0.137	1.000
Pyrexia	2 (1.3)	0 (0)	1 (1.5)	0 (0)	1 (1.2)	0 (0)	1.000	-
Alopecia	17 (11)	1 (0.6)	4 (5.9)	0 (0)	13 (15.1)	1 (1.2)	0.069	1.000
Hypothyroidism	17 (11)	0 (0)	7 (10.3)	0 (0)	10 (11.6)	0 (0)	0.997	-
Hyperthyroidism	3 (2)	0 (0)	2 (1.3)	0 (0)	1 (1.2)	0 (0)	0.904	-

“-” None value;

### Predictors of benefit from immune checkpoint inhibitors

The overall PFS and OS were 7.6 months (95% CI: 6.5–8.2) and 17.4 months (95% CI: 15.3–19.3), respectively.

#### Predictors of PFS

**Fig. 3:**
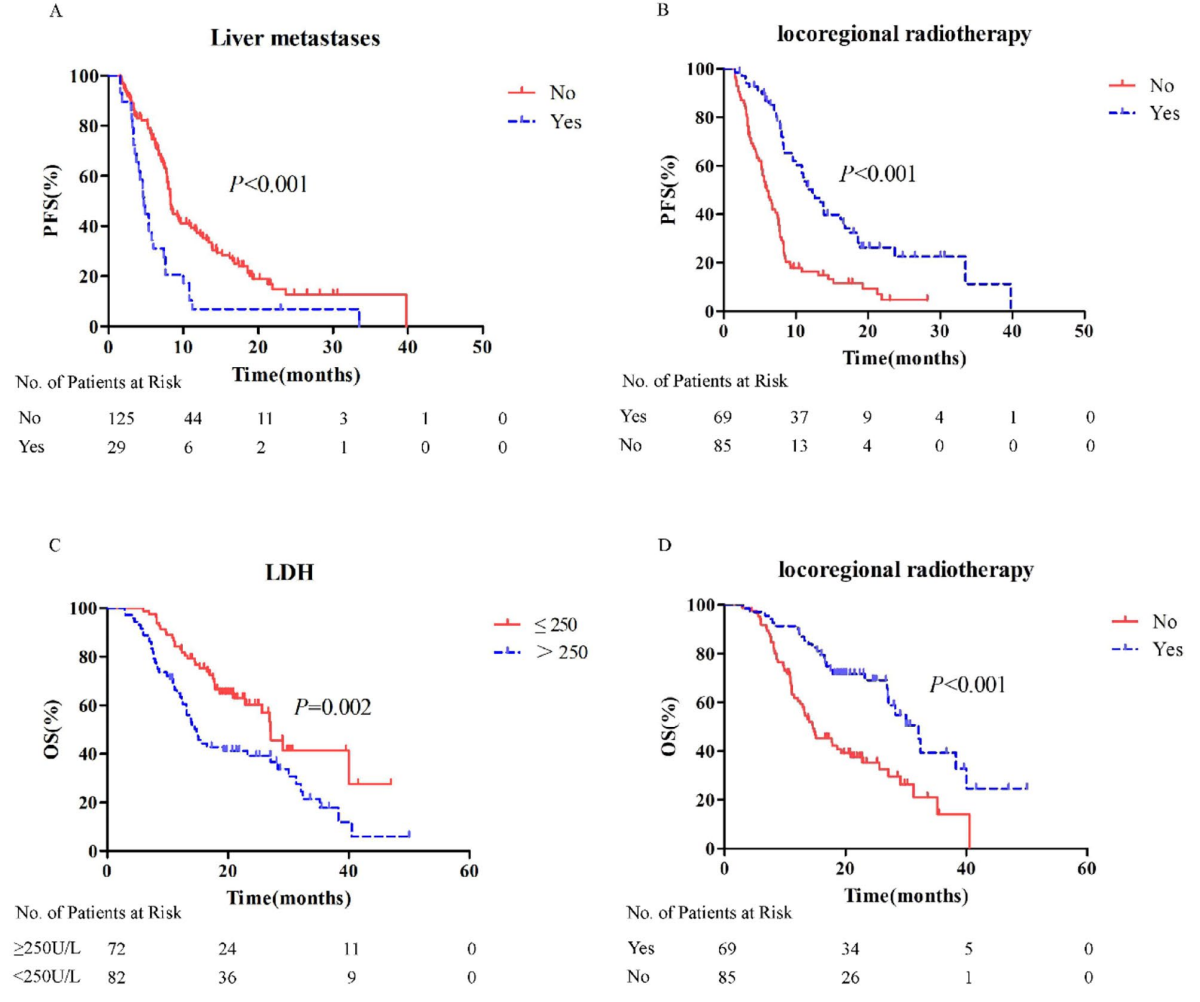
Predictors of PFS and OS in whole patients. **A**: Patients without liver metastases had longer PFS; **B**: Patients received locoregional thoracic radiotherapy had longer PFS; **C**: Patients with normal LDH had longer OS; **D**: Patients received locoregional thoracic radiotherapy had longer OS

In univariate analysis, liver metastases, bone metastases, ECOG-PS, locoregional thoracic radiotherapy, and LDH level were all significantly associated with PFS. In Cox regression analysis, only liver metastases and locoregional thoracic radiotherapy were independently associated with PFS (Table 4; Fig. 3A-B).

**Table 4:** Univariate analysis and Cox regression analysis of PFS and OS of all patients

Characteristics	PFS mPFS (95%CI)	*p* value	Cox regression		OS mOS (95%CI)	*p* value	Cox regression	
			HR(95%CI)	*p* value			HR(95%CI)	*p* value
Sex		0.187						
Male	7.8 (7.201–8.399)							
Female	13.9 (2.384–25.416)							
Age		0.455				0.066		
≤ 65	8.0 (7.465–8.535)				27.1 (24.519–29.681)			
> 65	7.7 (5.440–9.960)				16.5 (12.471–20.529)			
Liver metastases		0.000	1.862 (1.173–2.957)	0.008		0.000	1.547 (0.891–2.684)	0.121
No	8.3 (7.728–8.872)				27.1 (22.398–31.802)			
Yes	4.7 (3.997–5.403)				10.9 (8.966–12.834)			
Brain metastases		0.903				0.495		
No	7.8 (7.182–8.418)				23.2 (15.251–31.149)			
Yes	8.0 (7.388–8.612)				28.2 (17.998–38.402)			
Bone metastases		0.013	0.895 (0.570–1.404)	0.628		0.000	1.563 (0.939–2.603)	0.086
No	8.4 (7.293–9.507)				29.1 (24.633–33.567)			
Yes	5.3 (3.887–6.713)				11.1 (8.102–14.098)			
Smoking history		0.156				0.319		
No	8.4 (6.413–10.387)				26.9 (18.831–34.969)			
Yes	7.8 (6.938–8.662)				23.2 (13.258–33.142)			
ECOG PS		0.015	1.670 (0.943–2.958)	0.079		0.028	1.563 (0.813–3.004)	0.180
≤ 1	8.2 (7.670–8.730)				26.9 (21.758–32.042)			
> 1	5.8 (4.150–7.450)				10.8 (0.000-24.847)			
Chemotherapy regimen		0.505				0.861		
EP	8.2 (7.301–9.099)				22.8 (12.910–32.690)			
EC	7.8 (7.024–8.576)				25.6 (14.358–36.842)			
EL	8.2 (6.049–10.351)				27 (18.310–35.690)			
Locoregional thoracic radiotherapy		0.000	0.422 (0.28–0.635)	0.000		0.000	0.540 (0.333–0.878)	0.013
No	6.0 (4.857–7.143)				14.6 (10.51–18.690)			
Yes	12.3 (9.364–15.236)				32.1 (25.602–38.598)			
LDH		0.021	1.163 (0.790–1.711)	0.444		0.002	1.580 (1.006–2.482)	0.047
≤ 250	8.3 (7.505–9.095)				27.0 (23.664–30.336)			
>250	7.4 (6.161–8.639)				14.8 (12.409–17.191)			

mOFS: median progression free survival, mOS: median overall survival

#### Predictors of OS

In univariate analysis, liver metastases, bone metastases, ECOG-PS, locoregional thoracic radiotherapy, and LDH level were significantly associated with OS. In Cox regression analysis, only locoregional thoracic radiotherapy and LDH level were independently associated with OS (Table 4; Fig. 3C-D).

**Fig. 4:**
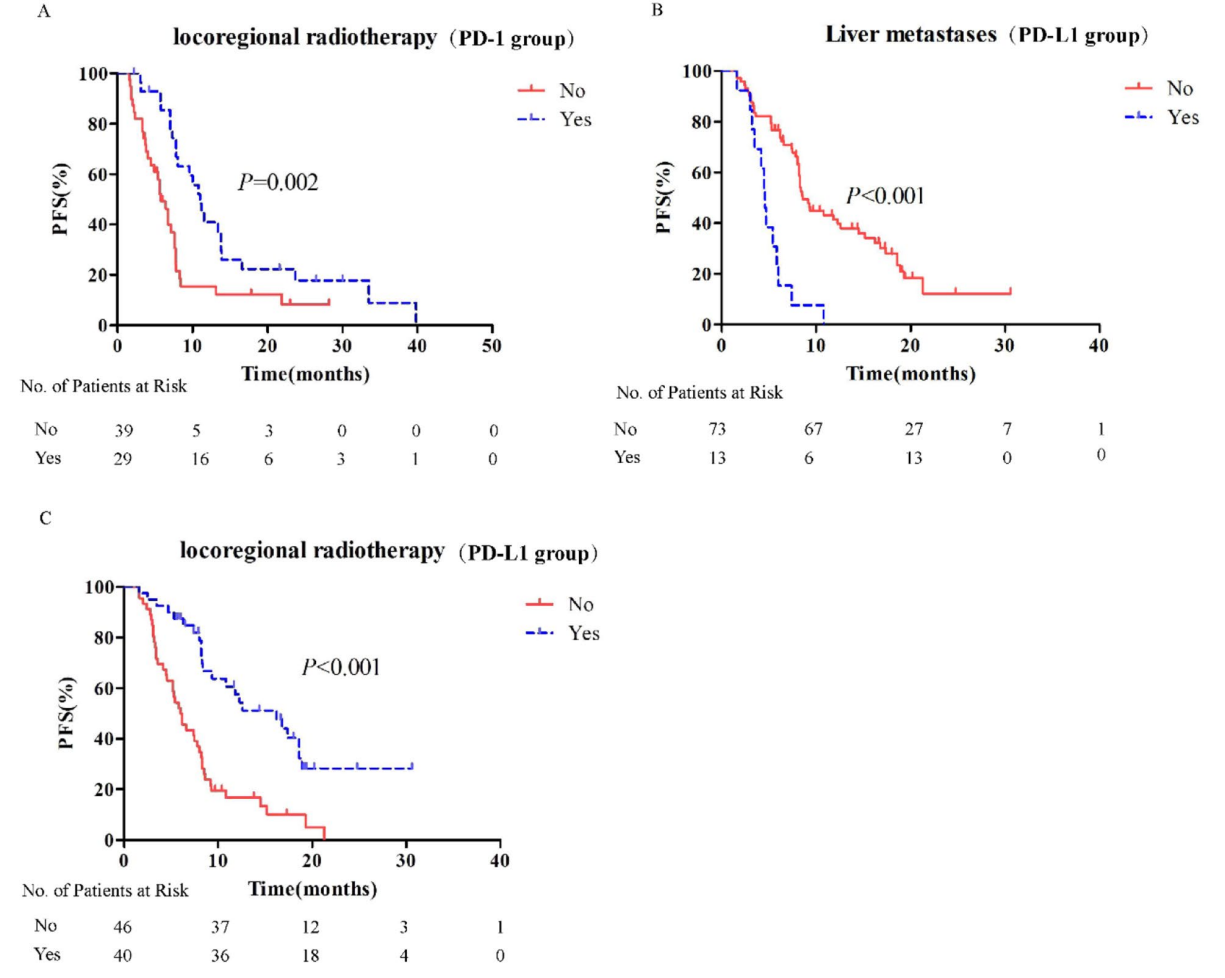
Stratified analysis of PFS in patients of PD-1 group or PD-L1 group. **A**: In PD-1 group, locoregional thoracic radiotherapy was related with better PFS; **B**-**C**: In PD-L1 group, liver metastases and locoregional thoracic radiotherapy were related with better PFS

Stratified analysis was performed to identify the characteristics of patients most likely to benefit in each treatment group. In the PD-1 group, univariate analysis showed that locoregional thoracic radiotherapy and LDH level were associated with PFS; however, in Cox regression analysis, only locoregional thoracic radiotherapy was an independent predictor of better PFS (Fig. 4A, Supplemental Tables 2–3). In the PD-L1 group, liver metastases, bone metastases, smoking history, and locoregional thoracic radiotherapy were related to PFS. In Cox regression analysis, only absence of liver metastases and receipt of locoregional thoracic radiotherapy were independent predictors of better PFS (Fig. 4B-C, Supplemental Tables 2–3). No clinical characteristic was found to be associated with OS benefit in patients treated with anti-PD-1 or anti-PD-L1 agents (Supplemental Tables 4–5).

## Discussion

Clinical trials have demonstrated differential efficacy of anti-PD-1 agents and anti-PD-L1 agents in first-line treatment of ES-SCLC patients [9–12, 14, 15]. Direct head-to-head trials comparing anti-PD-1 agents and anti-PD-L1 agents are difficult to conduct as clinical trials are expensive and time consuming, and require multiparty collaboration. The present study suggests that anti-PD-1 and anti-PD-L1 agents have similar efficacy and safety in ES-SCLC when delivered in combination with platinum-based doublet chemotherapy as first-line treatment.

A preclinical study has shown that anti-PD-L1 agents only inhibit PD-L1/PD-1 and PD-L1/B7-1 signaling and restore tumor-specific T-cell immunity [18], whereas anti-PD-1 agents block both PD-L1 and PD-L2 binding to PD-1 [19]. This suggests that anti-PD-1 agents might have stronger anti-tumor activity than anti-PD-L1 agents. Indeed, an indirect comparison meta-analysis found that the combination of chemotherapy plus anti-PD-1 agent provides better OS benefit than chemotherapy plus anti-PD-L1 agent in patients with NSCLC [20]. The immune microenvironment in SCLC is distinctly different from that in NSCLC, with PD-L1 expression being typically low or absent in the former. According to Wang et al., ES-SCLC is characterized with overexpression of cell cycle– and DNA repair–related genes, and decreased expression of immune-related genes [21]. Thus, the differential antitumor efficacy of anti-PD-1 agents and anti-PD-L1 agents may result in different outcomes in SCLC.

Gadgeel et al. reported that maintenance with pembrolizumab after induction chemotherapy in ES-SCLC showed no clinical benefit when compared with historical data [22]. In later-line treatment of SCLC, anti-PD-1 agents (pembrolizumab or nivolumab) as third- or later-line treatment provided duration of response of ~ 18 months and ORR of 12-33.3% [5, 23]. In comparison, with anti-PD-L1 agents (atezolizumab or durvalumab) as second-line or later treatment, the ORR was only 2.3% or 9.5% [24, 25]; however, median PFS and OS were 1.4-2.0 months and 5.6–7.7 months, respectively, for anti-PD-1 agents vs. 1.4–1.5 months and 4.8–9.5 months, respectively, for anti-PD-L1 agents. Moreover, both nivolumab and atezolizumab failed to improve OS compared with chemotherapy as second-line therapy in SCLC [24, 26]. These findings suggest higher response rates with anti-PD-1 agents than with anti-PD-L1 agents, but no survival advantage in later-line settings of SCLC. As in the first-line setting in ES-SCLC, a meta-analysis that indirectly compared the efficacy of anti-PD-1 agents plus chemotherapy to anti-PD-L1 agents plus chemotherapy found no significant difference between anti-PD-1 agents and anti-PD-L1 agents [16]. Our study also strongly suggests that anti-PD-1 agents and anti-PD-L1 agents have similar efficacy in ES-SCLC.

In terms of long-term survival, surprisingly, our results demonstrated the most remarkable overall survival than in previous clinical trials on ES-SCLC. There could be several possible explanations. First, 44.8% of patients in our study received locoregional thoracic radiotherapy, and this may have contributed to the better survival. The efficacy of locoregional thoracic radiotherapy in ES-SCLC patients treated with ICIs has not yet been studied thoroughly, but stratification analysis in our study found locoregional thoracic radiotherapy to be an independent predictor of OS and PFS. This is consistent with the retrospective study by Wu et al. [27].. While clinical trials such as IMpower-133 and CASPIAN did not allow locoregional radiotherapy, several other studies have consistently shown that thoracic radiotherapy after first-line chemotherapy can improve OS and PFS in ES-SCLC patients [28, 29]. Second, it might partially be due to large proportion of patients who received six cycles of chemotherapy plus ICIs. Only four cycles were administered in clinical trials such as KEYNOTE-604, IMpower-133, CASPIAN, and ASTRUM-005. Similar long OS was also observed in CAPSTONE-1, in which the patients received four to six cycles of chemotherapy plus anti-PD-L1 antibody. Third, ICIs exhibit durable antitumor efficacy in patients who show good initial response. In CheckMate-032 and KEYNOTE-028, the median duration of response to nivolumab or pembrolizumab among SCLC patients who showed good initial response was 17.9 months and 19.4 months, respectively [5, 6]. In our study, the initial response to ICI treatment was high (ORR, 79%) and better than in the above-mentioned clinical trials; this too may have contributed to the superior survival outcomes in our study. These possibilities need to be explored in further studies.

In stratified analysis, we found that ES-SCLC patients with normal LDH value benefit more from ICI than patients with abnormally high LDH levels; this is consistent with Lim et al. [30].. A previous study suggested that LDH might be an indicator of tumor burden and that increased LDH inhibits antitumor activity of immune checkpoints by altering metabolism, nutrient availability, and acidic microenvironment [31]. In CheckMate-331, among SCLC patients with below-normal LDH, OS tended to be longer in patients receiving nivolumab as second-line treatment than in patients receiving chemotherapy [26]. In CAPSTONE-1 also, among patients treated with anti-PD-L1 agent plus chemotherapy, subgroup analysis showed significantly better OS in patients with below-normal LDH than in patients with high LDH [12]. Thus, LDH is associated with OS in ES-SCLC patients treated with chemotherapy plus ICI and could be a predictive biomarker for identifying patients more likely to benefit from ICI in the first-line setting. However, further investigation is needed.

Platinum is the cornerstone of chemotherapy in ES-SCLC but is used differently in different regions and institutions. In most regions, carboplatin is generally preferred for SCLC in the first-line setting, with cisplatin being used only for 27-42% of patients [32]. We explored whether the type of platinum affected efficacy of ICI but found no difference in the clinical benefit of anti-PD-1/PD-L1 agents among patients treated with cisplatin, carboplatin or lobaplatin. CASPIAN, KEYNOTE-604, and EA-5161 also found that there was benefit from addition of ICI regardless of the platinum regimen.

The findings of this study suggest that anti-PD-1 agents and anti-PD-L1 agents have similar safety profiles. Myelosuppression, liver injury, and gastrointestinal reaction were the most common TRAEs; this is consistent with previous clinical trials. Notably, 9.7% and 6.5% of patients in our study experienced all-grade and ≥ grade 3 pneumonia; these rates are higher than those reported in previous clinical trials and were probably due to the high proportion of patients receiving locoregional thoracic radiotherapy in our study.

When interpretating our findings, some limitations should be noted. First, this was retrospective study with a relatively small sample size and insufficient statistical power; in addition, missing valuable was inevitable due to the nature of retrospective study. Second, as PD-L1 expression or tumor mutational burden (TMB) was not assessed in the majority of patients, the association between PD-L1 expression or TMB and efficacy of ICIs was not analyzed; however, previous clinical trials have found that the antitumor activity of ICIs in SCLC in the first- or later-line settings is not influenced by PD-L1 expression or TMB value [23, 26, 33].

In conclusion, anti-PD-1 and anti-PD-L1 agents combined with chemotherapy as first-line treatment in ES-SCLC appear to be comparably effective and well tolerated. The clinical benefit of ICI in ES-SCLC is evident regardless of the platinum regimen used. Addition of locoregional thoracic radiotherapy may further improve survival outcomes. Normal serum LDH level could be a useful biomarker to identify patients more likely to benefit from ICI. Future studies with larger sample size and direct comparison between anti-PD-1 agents and anti-PD-L1 agents are needed to confirm our findings.

### Electronic supplementary material

Below is the link to the electronic supplementary material.

**Supplementary Material 1: Supplemental Table 1.** Subgroup analysis of PFS and OS between PD-1 group and PD-L1 group. **Supplemental Table 2.** Univariate analysis of PFS in PD-1 group and PD-L1 group. **Supplemental Table 3.** Cox regression analysis of PFS in PD-1 group and PD-L1 group. **Supplemental Table 4.** Univariate analysis of OS in PD-1 group and PD-L1 group. **Supplemental Table 5.** Cox regression analysis of OS in PD-1 group and PD-L1 group

## Data Availability

The datasets analyzed during the current study available from the corresponding author on reasonable request.
