# Supplementary material for: Efficacy and safety of anti-PD-1 inhibitor versus anti-PD-L1 inhibitor in first-line treatment of extensive-stage small cell lung cancer: a multicenter retrospective study
Source: BMC Cancer. 2024 Jan 17;24:100. doi: 10.1186/s12885-024-11833-6 (PMC10795417; doi:10.1186/s12885-024-11833-6)
Supplement: Supplementary file 1 — Supplementary Material 1: Supplemental Table 1. Subgroup analysis of PFS and OS between PD-1 group and PD-L1 group. Supplemental Table 2. Univariate analysis of PFS in PD-1 group and PD-L1 group. Supplemental Table 3. Cox regression analysis of PFS in PD-1 group and PD-L1 group. Supplemental Table 4. Univariate analysis of OS in PD-1 group and PD-L1 group. Supplemental Table 5. Cox regression analysis of OS in PD-1 group and PD-L1 group [file 12885_2024_11833_MOESM1_ESM.docx]

**Supplemental Table 1. Subgroup analysis of PFS and OS between PD-1 group and PD-L1 group**

| Characteristics | PD-1 group  mPFS (95%CI) | PD-L1 group  mPFS (95%CI) | p value | PD-1 group  mOS (95%CI) | PD-L1 group  mOS (95%CI) | p value |
| --- | --- | --- | --- | --- | --- | --- |
| Age |  |  |  |  |  |  |
| ≤65 | 7.8 (6.892-8.708) | 8.2 (7.595-8.805) | 0.996 | 29.1 (23.333-34.867) | 27.0 (18.369-35.631) | 0.514 |
| >65 | 5.7 (5.299-6.101) | 8.3 (6.729-9.871) | 0.080 | 10.9 (8.405-13.395) | 19.3 (12.369-26.231) | 0.235 |
| Liver metastases |  |  |  |  |  |  |
| No | 7.8 (7.156-8.444) | 8.6 (7.571-9.629) | 0.312 | 29.1 (20.014-38.186) | 27.0 (22.150-31.850) | 0.632 |
| Yes | 4.9 (2.156-7.644) | 4.6 (4.013-5.187) | 0.148 | 10.9 (9.463-12.337) | 8.6 (2.024-15.176) | 0.609 |
| Brain metastases |  |  |  |  |  |  |
| No | 7.6 (6.189-9.011) | 8.3 (6.969-9.631) | 0.249 | 22.8 (7.968-37.632) | 23.2 (13.743-32.657) | 0.819 |
| Yes | 8.3 (3.72-12.88) | 8 (4.318-11.682) | 0.237 | 32.1 (6.925-57.275) | 27.0 (7.649-46.351) | 0.400 |
| Bone metastases |  |  |  |  |  |  |
| No | 7.8 (6.937-8.663) | 8.6 (7.653-9.547) | 0.484 | 32.1 (26.685-37.515) | 27.1 (20.487-33.713) | 0.484 |
| Yes | 5.6 (3.409-7.791) | 4.6 (3.817-5.383) | 0.877 | 10.9 (9.810-11.990) | 14.0 (6.124-21.876) | 0.593 |
| Smoking history |  |  |  |  |  |  |
| No | 7.6 (6.752-8.448) | 16.2 (6.189-26.211) | 0.047 | 22.8 (9.891-35.709) | 31.3 | 0.174 |
| Yes | 7.7 (6.733-8.667) | 8 (6.399-9.601) | 0.760 | 27.1 (3.560-50.64) | 17.9 (9.219-26.581) | 0.466 |
| chemotherapy regimen |  |  |  |  |  |  |
| EP | 7.8 (6.090-9.51) | 8.2 (7.009-9.391) | 0.942 | 22.8 (10.531-35.069) | 23.2 (13.582-32.818) | 0.954 |
| EC | 7.6 (6.792-8.408) | 8.3 (7.350-9.25) | 0.717 | 32.1 (8.684-55.516) | 18.6 (10.508-26.692) | 0.440 |
| EL | 6.7 (4.179-9.221) | 8.3 (0.000-21.546) | 0.175 | 13.2 (8.999-17.401) | 31.3 | 0.132 |
| Locoregional thoracic radiotherapy |  |  |  |  |  |  |
| No | 5.8 (4.623-6.977) | 6.0 (4.560-7.440) | 0.964 | 13.7 (8.962-18.438) | 15.1 (9.824-20.376) | 0.770 |
| Yes | 11 (8.970-13.030) | 16.2 (9.863-22.537) | 0.253 | 32.4 (24.005-40.795) | 28.2 (24.861-31.539) | 0.615 |
| LDH |  |  |  |  |  |  |
| ≤250 | 7.8 (5.102-10.498) | 8.4 (8.062-8.738) | 0.998 | 27.1 (19.867-34.333) | 27.0 (16.970-37.030) | 0.918 |
| ＞250 | 7.1 (5.286-8.914) | 8 (6.337-9.663) | 0.073 | 13.2 (9.872-16.528) | 15.6 (9.74-21.46) | 0.758 |

mOFS: median progression free survival, mOS: median overall survival.

**Supplemental Table 2. Univariate analysis of PFS in PD-1 group and PD-L1 group**

| Characteristics | PD-1 group  mPFS (95%CI) | p value | PD-L1 group  mPFS (95%CI) | p value |
| --- | --- | --- | --- | --- |
| sex |  | 0.243 |  | 0.445 |
| Male | 7.6 (6.902-8.298) |  | 8.2 (7.617-8.783) |  |
| Female | 13.9 (3.208-24.592) |  | 16.8 (0.000-37.542) |  |
| Age |  | 0.054 |  | 0.815 |
| ≤65 | 7.8 (6.892-8.708) |  | 8.2 (7.595-8.805) |  |
| >65 | 5.7 (5.299-6.101) |  | 8.3 (6.729-9.871) |  |
| Liver metastases |  | 0.153 |  | 0.000 |
| No | 7.8 (7.156-8.444) |  | 8.6 (7.571-9.629) |  |
| Yes | 4.9 (2.156-7.644) |  | 4.6 (4.013-5.187) |  |
| Brain metastases |  | 0.250 |  | 0.166 |
| No | 7.6 (6.189-9.011) |  | 8.3 (6.969-9.631) |  |
| Yes | 8.3 (3.720-12.88) |  | 8.0 (4.318-11.682) |  |
| Bone metastases |  | 0.144 |  | 0.046 |
| No | 7.8 (6.937-8.663) |  | 8.6 (7.653-9.547) |  |
| Yes | 5.6 (3.409-7.791) |  | 4.6 (3.817-5.383) |  |
| Smoking history |  | 0.756 |  | 0.024 |
| No | 7.6 (6.752-8.448) |  | 16.2 (6.189-26.211) |  |
| Yes | 7.7 (6.733-8.667) |  | 8.0 (6.399-9.601) |  |
| chemotherapy regimen |  | 0.588 |  | 0.276 |
| EP | 7.8 (6.090-9.510) |  | 8.2 (7.009-9.391) |  |
| EC | 7.6 (6.792-8.408) |  | 8.3 (7.350-9.250) |  |
| EL | 6.7 (4.179-9.221) |  | 8.3 (0.00-21.546) |  |
| Locoregional thoracic radiotherapy |  | 0.002 |  | 0.000 |
| No | 5.8 (4.623-6.977) |  | 6.0 (4.560-7.440) |  |
| Yes | 11.0 (8.970-13.030) |  | 16.2 (9.863-22.537) |  |
| LDH |  | 0.019 |  | 0.336 |
| ≤250 | 7.8 (5.102-10.498) |  | 8.0 (6.337-9.663) |  |
| ＞250 | 7.1 (5.286-8.914) |  | 8.3 (7.717-8.883) |  |

mPFS: median progression free survival.

**Supplemental Table 3. Cox regression analysis of PFS in PD-1 group and PD-L1 group**

|  | PD-1 group | |  | PD-L1 group | |
| --- | --- | --- | --- | --- | --- |
|  | HR（95%CI） | p value |  | HR（95%CI） | p value |
| Liver metastases |  |  |  | 2.281 (1.056-4.925) | 0.036 |
| Bone metastases |  |  |  | 1.093 (0.584-2.044) | 0.781 |
| Smoking history |  |  |  | 1.625 (0.859-3.073) | 0.136 |
| Locoregional thoracic radiotherapy | 0.483 (0.271-0.858) | 0.013 |  | 0.416 (0.238-0.726) | 0.002 |
| LDH | 1.603 (0.905-2.840) | 0.106 |  |  |  |

HR: hazard ratio

**Supplemental Table 4. Univariate analysis of OS in PD-1 group and PD-L1 group**.

| Characteristics | PD-1 group  mOS (95%CI) | p value | PD-L1 group  mOS (95%CI) | p value |
| --- | --- | --- | --- | --- |
| sex |  | 0.051 |  |  |
| Male | 15.3 (7.431-23.169) |  |  |  |
| Female | 26.9 (20.762-33.038) |  |  |  |
| Age |  | 0.043 |  | 0.519 |
| ≤65 | 29.1 (23.333-34.867) |  | 27.0 (18.369-35.631) |  |
| >65 | 10.9 (8.405-13.395) |  | 19.3 (12.369-26.231) |  |
| Liver metastases |  | 0.049 |  | 0.000 |
| No | 29.1 (20.014-38.186) |  | 27.0 (22.150-31.850) |  |
| Yes | 10.9 (9.463-12.337) |  | 8.6 (2.024-15.176) |  |
| Brain metastases |  | 0.509 |  | 0.938 |
| No | 22.8 (7.968-37.632) |  | 23.2 (13.743-32.657) |  |
| Yes | 32.1 (6.925-57.275) |  | 27.0 (7.649-46.351) |  |
| Bone metastases |  | 0.001 |  | 0.010 |
| No | 32.1 (26.685-37.515) |  | 27.1 (20.487-33.713) |  |
| Yes | 10.9 (9.810-11.99) |  | 14.0 (6.124-21.876) |  |
| Smoking history |  | 0.842 |  |  |
| No | 22.8 (9.891-35.709) |  |  |  |
| Yes | 27.1 (3.560-50.64) |  |  |  |
| ECOG PS |  | 0.147 |  | 0.112 |
| ≤1 | 26.9 (13.673-40.127) |  | 27 (18.853-35.147) |  |
| >1 | 8.3 (0.000-17.326) |  | 10.8 (0.000-23.689) |  |
| chemotherapy regimen |  | 0.633 |  |  |
| EP | 22.8 (10.531-35.069) |  |  |  |
| EC | 32.1 (8.684-55.516) |  |  |  |
| EL | 13.2 (8.999-17.401) |  |  |  |
| Locoregional thoracic radiotherapy |  | 0.005 |  | 0.004 |
| No | 13.7 (8.962-18.438) |  | 15.1 (9.824-20.376) |  |
| Yes | 32.4 (24.005-40.795) |  | 28.2 (24.861-31.539) |  |
| LDH |  | 0.017 |  | 0.058 |
| ≤250 | 27.1 (19.867-34.333) |  | 27 (16.970-37.030) |  |
| >250 | 13.2 (9.872-16.528) |  | 15.6 (9.740-21.460) |  |

mOS: median overall survival

**Supplemental Table 5. Cox regression analysis of OS in PD-1 group and PD-L1 group**

|  | PD-1 group | |  | PD-L1 group | |
| --- | --- | --- | --- | --- | --- |
|  | HR（95%CI） | p value |  | HR（95%CI） | p value |
| Age | 2.013 (0.994-4.078) | 0.052 |  |  |  |
| Liver metastases | 1.099 (0.491-2.462) | 0.818 |  | 1.908 (0.838-4.341) | 0.124 |
| Bone metastases | 2.309 (0.968-5.508) | 0.059 |  | 1.543 (0.776-3.068) | 0.216 |
| Locoregional thoracic radiotherapy | 0.554 (0.258-1.193) | 0.132 |  | 0.541 (0.274-1.069) | 0.077 |
| LDH | 1.515 (0.784-2.928) | 0.217 |  |  |  |

HR: hazard ratio
